# Depth-resolved rhodopsin molecular contrast imaging for functional assessment of photoreceptors

**DOI:** 10.1038/srep13992

**Published:** 2015-09-11

**Authors:** Tan Liu, Rong Wen, Byron L. Lam, Carmen A. Puliafito, Shuliang Jiao

**Affiliations:** 1 Department of Biomedical Engineering, Florida International University, Miami, Florida 33174, USA; 2 Bascom Palmer Eye Institute, Miller School of Medicine, University of Miami, Miami, Florida 33136, USA; 3 Keck School of Medicine, University of Southern California, Los Angeles, California 90089, USA

## Abstract

Rhodopsin, the light-sensing molecule in the outer segments of rod photoreceptors, is responsible for converting light into neuronal signals in a process known as phototransduction. Rhodopsin is thus a functional biomarker for rod photoreceptors. Here we report a novel technology based on visible-light optical coherence tomography (VIS-OCT) for *in vivo* molecular imaging of rhodopsin. The depth resolution of OCT allows the visualization of the location where the change of optical absorption occurs and provides a potentially accurate assessment of rhodopsin content by segmentation of the image at the location. Rhodopsin OCT can be used to quantitatively image rhodopsin distribution and thus assess the distribution of functional rod photoreceptors in the retina. Rhodopsin OCT can bring significant impact into ophthalmic clinics by providing a tool for the diagnosis and severity assessment of a variety of retinal conditions.

Rhodopsin is the light-sensing molecule in rod photoreceptors with 11-*cis*-retinaldehyde (retinal) as the chemical basis of light sensitivity and rod opsin protein as the signaling basis. Upon photon absorption, the retinal isomerizes to the all-*trans* state, which triggers the phototransduction cascade^1^. The isomerization of 11-*cis*-retinal to the all-*trans* leads to a dramatic shift in the absorption spectrum: the absorption peak (λ_peak_) shifts from 500 nm to 380 nm^2^,^3^,^4^. This light-induced shift of rhodopsin absorption spectrum, known as rhodopsin photo-bleaching, has been used to assess rhodopsin content by retinal densitometry or optical reflectometry since the 1950s^5^,^6^,^7^. The same principle^8^ also provides a contrast for imaging rhodopsin, which has been verified with imaging technologies like fundus photography^9^,^10^,^11^ and scanning laser ophthalmoscopy (SLO)^12^,^13^,^14^,^15^. Because all these imaging technologies have no depth resolving capability, their images are constructed with photons reflected from tissues in the entire imaging depth. In addition, the theoretical models on fundus reflection are based on hypothetical pathways of how photons propagate in the retina. The available rhodopsin imaging techniques can provide neither experimental data to directly verify the proposed theoretical models nor depth resolved visualization of the distribution of light absorption^12^,^16^. Three-dimensional imaging using a wavelength close to the rhodopsin λ_peak_ should not only provide more insight into the distribution of absorption along the depth and thus the photon propagation pathways, but also accurately quantify rhodopsin distributions in the retina. This can be achieved by spectral domain optical coherence tomography (SD-OCT)^17^,^18^ using a light source with a center wavelength close to the rhodopsin λ_peak._

SD-OCT has been widely used for ophthalmic imaging in both clinical diagnosis and laboratory studies due to its high depth resolving capability^19^,^20^. Different contrasts for OCT have been explored to image the different properties of biological tissues, including the scattering contrast for structural imaging, polarization contrast for tissue birefringence imaging, Doppler-effect related contrasts for blood flow and vasculature imaging, spectroscopic molecular contrast for imaging endogenous and exogenous chromophores in the blood vessels^21^, and hemoglobin-specific contrast for retinal blood vessel oximetry^22^.

Here we report a novel technology based on visible-light optical coherence tomography (VIS-OCT) for *in vivo* molecular imaging of rhodopsin. The technique was successfully tested on animals *in vivo*.

## Results

### System performance

**Figure 1:**
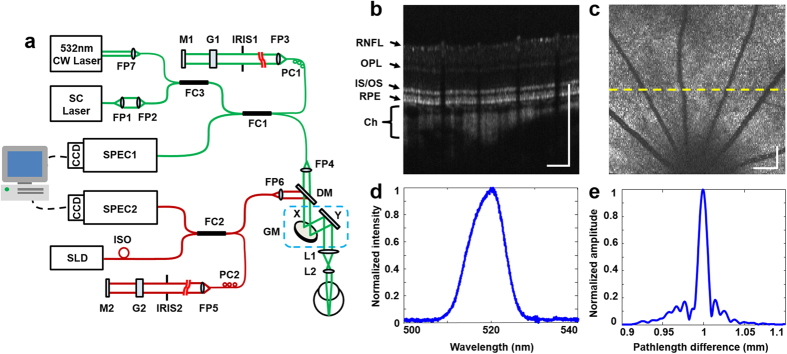
Imaging system and its performance. (**a**) Schematic of the fiber-based OCT system. A supercontiuumm laser (SC) and a superluminscent diode (SLD) are used as the light sources of the VIS- (green lines) and NIR-OCT (red lines) subsystems. A 532 nm continuous-wave laser (CW) is used as pattern bleaching light source. The light in the sample arms of the two OCTs is combined by a dichroic mirror (DM), scanned together by a galvanometer scanner (GM), and delivered to the retina by a pair of lenses (L1 and L2). The OCT signals are detected in the spectral domain by the two corresponding spectrometers (SPEC1-2). FP1-7: Fiber collimating port; ISO: Isolator; FC1-3: 3dB fiber coupler; PC1-2: Polarization controller; G1-2: BK-7 glass plate; M1-2: Mirror; IRIS1-2: Iris. (**b**) VIS-OCT cross-sectional image displayed in linear scale. RNFL: retinal nerve fiber layer; OPL: outer plexiform layer; IS/OS: junction between the inner and outer segments of the photoreceptors; RPE: retinal pigment epithelium; Ch: choroid; (**c**) VIS-OCT fundus image of a rat retina generated by projecting the 3D OCT data onto the *x-y* plane, in which the dotted line marks the location of (**b**); (**d**) spectrum of the VIS-OCT; (**e**) measured point spread function (PSF) of the VIS-OCT. Bar: 250 μm.

We designed and built a 3D retinal densitometry system based on VIS-OCT to image the molecular contrast of rhodopsin (Fig. 1a). The system uses a supercontinuum light source with a tunable filter. The filtered output has a center wavelength of 520 nm and a bandwidth of 9.3 nm (Fig. 1e), which enables a depth resolution of 13.7 μm in air (Fig. 1f) and about 9.8 μm in biological tissues assuming a refractive index of 1.4. A near infrared (NIR) OCT working at 840 nm is integrated into the system to assist alignment in dark without bleaching rhodopsin (Fig. 1a). The NIR- and VIS-OCT probe light beams are coaxial and delivered to the eye by the same scanning and imaging optics to assure they image the same area of the retina. The VIS-OCT is capable of acquiring 3D OCT dataset at a line rate of 64k lines/s. The performance of the system can be demonstrated with an acquired VIS-OCT 3D dataset of a rat retina represented either in a high-density cross-sectional image (B-scan, Fig. 1b) or a projected OCT fundus image (Fig. 1c). All the major retinal layers can be recognized in the cross-sectional image.

### *In vivo* animal imaging

**Figure 2:**
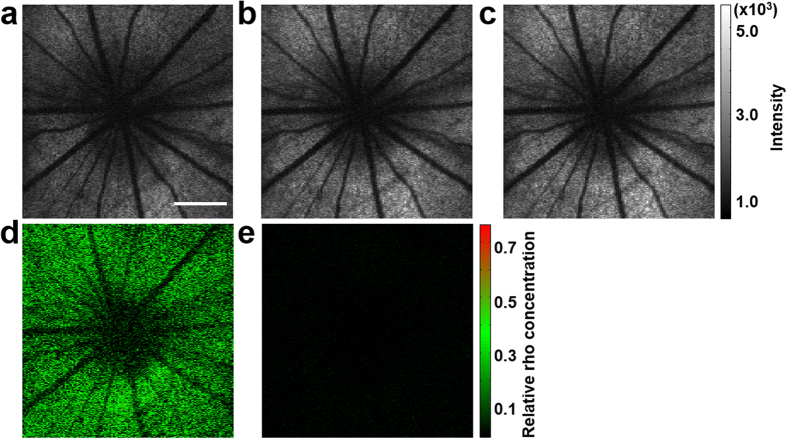
*En face* representation of rhodopsin imaging of an albino rat retina displayed in the *x-y* plane. All the images were generated by summing the signal intensities from IS/OS forward along the z direction. (**a**) dark-adapted image; (**b**) 1^st^ light-adapted image; (**c**) 2^nd^ light-adapted image acquired immediately after (**b**); (**d**) Differential image calculated between (**a**,**b**) using Eq. (1); (**e**) Differential image between (**b**,**c**) calculated with the same formula. (**a**–**c**) share the same color map. (**d**,**e**) also share the same color map. Bar: 500 μm.

We used Sprague Dawley rats to image rhodopsin and to investigate how photons propagate in retinal tissues in the depth direction. The animal was first dark adapted for 4 hours^23^. The retinal area of interest was located and the retinal image was optimized with the NIR-OCT, which was then turned off and the VIS-OCT was triggered to acquire a full 3D OCT dataset. The acquisition time is 1 second for an imaging volume containing 512 × 128 depth scans (A-lines). Rhodopsin was then bleached by bright room light (~800 lx) for 15 seconds, followed by acquisition of two light-adapted 3D images of the same retinal area. We manually segmented each OCT cross-sectional image along the junction between the inner and outer segments (IS/OS) of the photoreceptors. An *en face* view of the segmented OCT data was generated by summing the signal intensities from the IS/OS forward along the depth direction (Fig. 2). In most of the imaged area, the pixel intensities in the dark-adapted image are weaker than those of the same area in the two sequentially acquired light-adapted images (Fig. 2b,c). This is clearly shown in the differential image (Fig. 2d) between the images of Fig. 2a,b, calculated with


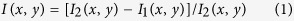


where *I*_1_ and *I*_2_ are the pixel intensity of the dark- and light-adapted images, respectively, *x* and *y* are the lateral coordinates of the images. The signals in Fig. 2d distributes evenly across the imaged area except at the optic disc where there was no photoreceptors. A differential image between the two light-adapted images in Fig. 2b,c was calculated with the same formula, where *I*_1_ and *I*_2_ are the pixel intensity of the first and second light-adapted images. As shown in Fig. 2e, the pixel intensities in the differential image between the two light-adapted images are less than 5% of those in the differential image (Fig. 2d) between the dark- and light-adapted images (Fig. 2d,e are displayed with the same color-map), indicating that the light reflection from retina in the light-adapted states is constant and stable. These results demonstrate that the signals in Fig. 2d represent light absorption by rhodopsin and thus the system is capable of rhodopsin imaging.

**Figure 3:**
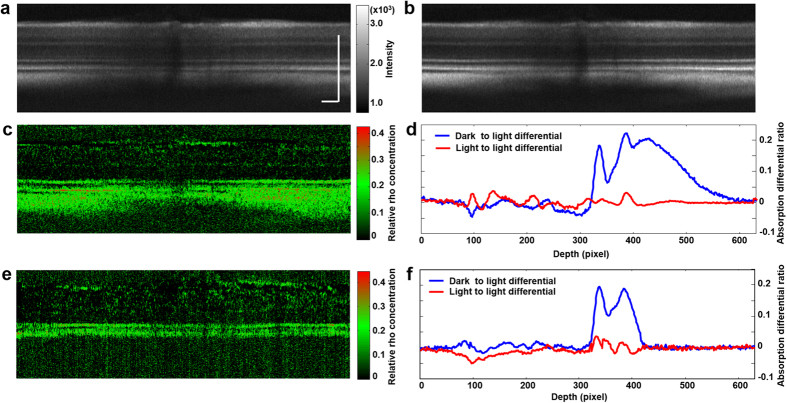
Depth-resolved rhodopsin imaging of albino and pigmented rat retinas displayed in the *x-z* plane. (**a**) Averaged cross-sectional image from all the B-scans of the dataset of the dark-adapted retina shown in Fig. 2(a); (b) Averaged cross-sectional image from all the B-scans of the dataset of the light-adapted retina shown in Fig. 2(b); (c) Differential image between (**a**,**b**) calculated with Eq. (2); (**d**) Averaged A-lines from the light to dark (blue line) and the light to light (red line) differential images for the albino rat; (**e**) Averaged cross-sectional differential image calculated between dark- and light- adapted states of a pigmented rat retina using Eq. (2); (**f**) Averaged A-lines from the light to dark (blue line) and the light to light (red line) differential images for the pigmented rat. (**a**,**b**) share the same color map. Bar: 100 μm.

To investigate how the reflections from different retinal layers contribute to the differential image of Fig. 2c, we averaged the respective 128 OCT B-scans in the 3D datasets of dark- and light-adapted states to suppress speckle. Before averaging, we shifted the A-lines in each OCT B-scan image in the depth direction in reference to the IS/OS, so that the IS/OS became a straight line. All the rearranged B-scan images were then aligned also in reference to the IS/OS and averaged (see Supplementary information A for details). Figure 3a,b show the averaged B-scan images in the dark- and light-adapted states, respectively. Figure 3c shows the differential image calculated using


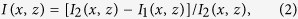


the same formula as that for Fig. 2d except that the image is displayed in the *x*-*z* plane, where *z* represents coordinates in the depth direction. The image (Fig. 3c) clearly shows that signals come from the layers corresponding to the photoreceptors, the retinal pigment epithelium (RPE), and the choroid. The residual high signal from some spots in the retinal nerve fiber layer (RNFL) is likely caused by slight misalignment between Fig. 3a,b and insufficient speckle cancellation. The relatively weaker signal in the central region is caused by the optic disc, where photoreceptors are absent and the retina curves the most. To further elucidate the changes in signals from different retinal layers between light- and dark-adapted states, we averaged the A-lines of Fig. 3c (Fig. 3d, blue line). Peaks of absorption change are seen in the photoreceptor layer, the RPE layer, and the choroid. In comparison, the averaged A-lines of the differential image between the two sequentially acquired light-adapted OCT datasets (Fig. 2b,c) showed no significant absorption changes (Fig. 3d, red line). The lowered signals from the retinal layers behind the photoreceptors in the dark-adapted state are also caused by rhodopsin absorption, similar to a shadowing effect caused by the photoreceptors. The probe light has to pass the photoreceptor layer to reach the RPE and choroid before being reflected. The reflected light again has to pass the photoreceptor layer before reaching the OCT interferometer. The light signals thus were attenuated by rhodopsin in either forward or return path.

In pigmented animals, the RPE cells and the choroid contain light-absorbing melanin. To investigate how the presence of melanin in the RPE cells and choroid would affect light reflection, we imaged the retina of Long Evans rat with the same procedure for the albino rats. The depth-resolved differential image between the averaged OCT B-scans of the light and dark adapted states is shown in Fig. 3e, and the averaged A-lines of the differential image is shown in Fig. 3f. It is clear that in the pigmented animal, the photoreceptor layer and the RPE contribute to the difference of fundus reflections between the two states of adaptation. The contribution from the choroid seen in the albino animal is absent because of the strong optical absorption of melanin that absorbs both the incident and the scattered light. Thus for pigmented animals, light reflected from behind the RPE can be neglected when quantifying rhodopsin from the measured fundus reflections.

### Pattern bleaching

**Figure 4:**
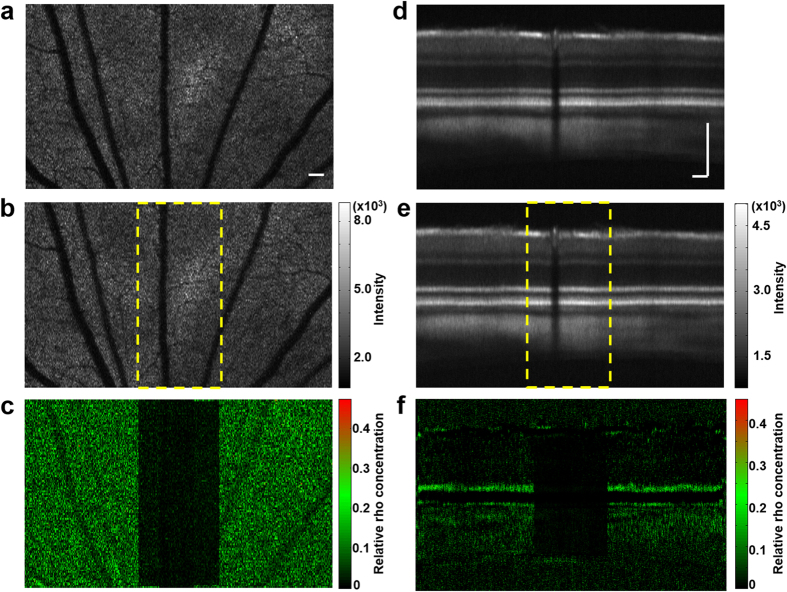
Rhodopsin imaging of albino rat retina with pattern bleach. (**a**) *En face* view of the OCT dataset of the dark-adapted retina where the vertical strip marked with the dotted yellow lines in Fig. 4(b) had been bleached with a 532 nm laser; (**b**) *En face* view of the OCT dataset of the light-adapted retina; (**c**) Differential image between (**a**,**b**) calculated with the same method as Fig. 2(c); (d) Averaged cross-sectional image of the same dark-adapted retina as (**a**); (**e**) Averaged cross-sectional image of the same light adapted retina as (**b**); (**f**) Differential image between (**d**,**e**) calculated with the same method as Fig. 3(c) (a,b) share the same color map, (d,e) also share the same color map. Bar: 100 μm.

To further verify that the differential images in Figs 2 and 3 represent the rhodopsin distribution, we designed a pattern bleaching experiment in which a vertical strip of the retina was pre-bleached by using a CW laser at 532 nm coupled into the optical fiber in the source arm of the OCT system. The patterned area was raster scanned twice consecutively with the bleaching laser at a scanning speed of 10k points/s. The power of the bleaching laser before entering the eye was 800 μW resulting in a total light energy of 160 nJ per illuminating point. In this experiment, the animal was first dark-adapted and the retinal image was optimized by using the NIR-OCT. The vertical strip of the retina was bleached, followed by acquisition of a dark-adapted 3D VIS-OCT dataset (Fig. 4a shows the projection of the dataset on the *x-y* plane). Then, after 15 seconds light adaption, a light-adapted VIS-OCT dataset was acquired (Fig. 4b shows the projection of the dataset on the *x-y* plane). The differential image (Fig. 4c), calculated with the same method as in Fig. 2, shows clearly a vertical stripe of no absorption, which corresponds well to the pre-bleached pattern (area between the two yellow lines, Fig. 4b). We further constructed a differential OCT B-scan image (Fig. 4f) using the same process as in Fig. 3. A strip of no absorption corresponding to the pre-bleached stripe is clearly seen in the photoreceptor layer, the RPE, and the choroid. Because rhodopsin in the photoreceptors is the only molecule bleachable at the wavelength and exposure level used, these results proved conclusively that the differential image represents rhodopsin distribution and the signals from the RPE and choroid in the differential image are also the results from rhodopsin absorption.

The regional pixel intensities calculated from the image in Fig. 4f are shown in Table 1. The mean pixel intensities (±standard deviation) in the inner retina in the pre-bleached strip are about 3% lower than that outside the strip, which is negligible. In contrast, in the photoreceptor layer the mean pixel intensities are significantly different in and outside the pre-bleached strip. Although 3% is considered insignificant, the lowered intensity values inside the pattern give rise to an appearance in Fig. 4f that the inner retina also has a bleaching effect. We speculate that this apparent bleaching effect could be caused by the different responses to the VIS-OCT probe light between the pre-bleached area and the rest of the retina. When the probe light was scanned inside the pre-bleached area there were little neuronal responses because rhodopsin had been bleached. When the probe light was scanned across rest of the retina neuronal responses could be stimulated, which can induce slight eye movement too small to be sensed by the imaging system but enough to change the speckle pattern of the OCT image outside the pre-bleached area. As a result, the effect of averaging on speckles may be different in and outside the bleached pattern and cause an apparent difference in the differential image.

## Discussion

We have demonstrated a VIS-OCT technology that is capable of imaging rhodopsin in the retina based on the principle of detecting light-induced rhodopsin absorption change, the same principle for fundus reflection densitometry or fundus reflectometry. The fundamental difference between this technology and all other fundus reflectometry technologies is that only this technology provides depth resolution. Depth information is critical for providing experimental data to test the theoretical models of photon propagation in the retina. For example, reflection from the sclera was previously included in the theoretical models^14^,^24^; our data, however, showed no sclera contribution although sclera is in the effective imaging range of the OCT system (See Supplementary information B and C for details).

The depth information is also essential to calculate rhodopsin concentration in the retina accurately. It has been proposed by van de Kraats *et al.*^14^,^24^ that light reflected from the retina is composed of reflections from three distinct layers: the ocular media anterior to the rod inner segment, where wave guiding by photoreceptors begins; the photoreceptor layer, where reflection by the outer segments and absorption by the bleachable visual pigment occurs; and the post-receptor layer, including the RPE, choroid, and sclera. With the depth-resolved OCT images we can get the reflections from all three different layers described in the model by segmentation and remove contributions from the anterior and post-receptor layers to allow more accurate quantification of rhodopsin concentration regardless of the degree of pigmentation in the eye. As shown clearly in our experiments with albino and pigmented animals that the degree of pigmentation of the post-receptor layer significantly influences contribution of that layer to fundus reflections. With segmentation the influence of reflection and attenuation of the probe light by tissues anterior to the photoreceptor layer on the quantification of rhodopsin can be eliminated.

Speckle is the major hurdle for the technology to display rhodopsin distribution in 3D. Summation or averaging is currently the most effective method for suppressing speckle at a cost of imaging time or spatial resolution. Our results show that calculating the rhodopsin absorption using Eq. (1) and Eq. (2) with the summed signals across the segmented photoreceptor layer and pre-photoreceptor layers speckle can be effectively managed. When we quantify rhodopsin in the depth-resolved B-scan images and don’t want to sacrifice the spatial resolution by averaging the pixels we must find a way to match the speckle pattern among the paired OCT images. We are currently working on testing different speckle suppressing techniques to achieve 3D display of rhodopsin distribution.

The VIS-OCT technology for rhodopsin imaging has potentially significant clinical impact. It can be used to construct an accurate topographic distribution of rhodopsin in the retina, i.e. a rhodopsin map. Since rhodopsin is a functional biomarker of rod photoreceptors, a rhodopsin map reflects the distribution of functional rod photoreceptors in the retina. Such information could be of clinical value in the diagnosis, longitudinal monitoring of disease progression and efficacy evaluation of treatments in retinal disorders with altered rhodopsin expression in photoreceptors or actual photoreceptor loss. For example, the progressive loss of photoreceptors in patients with hereditary retinal degenerations can be objectively measured and documented for clinical care and evaluation of treatments. Hereditary retinal degenerations are one of the major causes of blindness, including retinitis pigmentosa (RP), a group of genetically heterogeneous photoreceptor degenerative disorders with the prevalence of 1 in 3,000–4,500 people^25^,^26^,^27^,^28^. Detection of rod photoreceptor loss could also be of importance for the early diagnosis of age-related macular degeneration (AMD). Recent findings indicate that degeneration of rod photoreceptors exceeds the loss of cone photoreceptors in AMD^29^. The rapid development in regenerative medicine to restore vision has raised a hope that regeneration of photoreceptors and restoration of photoreceptor function will become reality in the near future. Accurate measurement of rhodopsin in the retina would be of particular value in the assessment of regenerative therapies aimed at restoration of photoreceptors and vision, including transplant stem cell-derived photoreceptors, gene therapies, neuroprotection therapies using neurotrophic factors and other neuroprotective agents.

When imaging human the technology needs to be as patient friendly as possible. Fixation light for stabilizing the eye by providing a gazing target needs to be in a wavelength longer than 650 nm to avoid bleaching of rhodopsin in imaging the dark-adapted retina. Eye tracking techniques may be needed to help reduce possible eye movement effect during imaging of both the dark- and light-adapted retina because visible light may cause eye movement more easily than NIR light. The light power of 240 μW is well below the ANSI safety limit, which poses no safety concerns. Light adaptation of the patient eye can be achieved with bright room light, which will not cause any discomfort. The current 1 second imaging time for acquiring the 3D OCT data can also be improved by using a faster line-scan camera in the spectrometer, which can help reduce the possible discomfort and eye movement caused by visible light illumination.

In summary, we have developed a VIS-OCT technology to accurately measure rhodopsin distribution and to functionally imaging rod photoreceptors in the retina. This technology could be used clinically to monitor the functional status of photoreceptors in the retina, for diagnosis and disease progression assessment in retinal degenerative disorders, including hereditary retinal degeneration and, perhaps more significantly, for the disease monitoring of dry AMD. The new technologies can also be used to measure the outcomes for therapeutic interventions aimed at preventing further photoreceptor loss, or restoration of photoreceptor numbers and function.

## Methods

### Experimental system

A schematic of the imaging system is shown in Fig. 1a. The system consisted of two independent SD-OCT subsystems, one of which worked in the NIR for guiding the alignment process and the other one worked in the visible for rhodopsin imaging. The VIS-OCT used a supercontinuum laser source (EXB-6, SuperK EXTREME, NKT Photonics, Denmark) equipped with a variable bandpass filter (SuperK Varia, NKT Photonics). The filtered output light (center wavelength: 520 nm, bandwidth: 9.3 nm, 80 MHz pulse rate) was delivered though a fiber delivery module. The VIS light was coupled into the source arm of a single-mode optical fiber-based Michelson interferometer by using a pair of identical fiber ports (PAF-X-11-A, Thorlabs). The NIR-OCT used a superluminescent diode (SLD-37-HP, center wavelength: 840 nm, bandwidth: 50 nm, Superlum, Russia). After passing through an optical fiber isolator the NIR light was coupled into another single-mode fiber-based Michaelson interferometer. After exiting their corresponding optical fibers in the sample arms the visible and NIR light beams were collimated and combined by a dichroic mirror. The combined light beam was scanned by a *x-y* galvanometer (6215H, Cambridge) scanner, and then delivered into the eye by the combination of a relay lens (f = 75 mm) and a 60D Volk lens. The VIS light power was 240 μW before entering the eye, while the NIR light at the same location was 600 μW. The theoretical axial resolution of the VIS-OCT was 12.5 μm in air and was measured to be 13.7 μm. The measured axial resolution of the NIR-OCT was 6.9 μm in air^30^. The lateral resolutions of both OCT subsystems were estimated to be around 20 μm, which were mainly limited by the optical aberrations of the eye.

In the detection arm of each OCT subsystem the reflected light from the sample and reference arms was collimated and detected by a spectrometer. The VIS-OCT spectrometer consists of an 1800 lines/mm transmission grating, a multi-element imaging lens (f = 150 mm), and a line scan CMOS camera (Sprint spL2048-70k, 2048 pixels with 10 μm pixel size; Basler, Ahrensburg, Germany). The NIR-OCT used a spectrometer with the same parameters as described in our previous publications^30^. In short, the NIR OCT used a line scan CCD camera (AVIIVA EM4 2k 4 × 12bits, 2048 pixels with 14 μm pixel size; e2V, Saint Egreve, France) working at a line rate of 24k lines/s. Two image acquisition boards (NI IMAQ PCIe-1433 for VIS-OCT and PCIe-1429 for NIR-OCT) acquired the interference spectra captured by the cameras and streamed them to a computer workstation for data processing. Spectrometer sensitivity falloff over imaging depth was measured and compensated before data analysis. The exposure time of the camera of the VIS-OCT was set to 11 μs. The camera was synchronized by the sampling clock of an analogue output board (PCI-6731, National Instruments) whose output controlled the scanning of the galvanometer scanner. The A-line rate was set to 64k lines/s.

In the rhodopsin mapping experiments, we used a raster scanning pattern to cover a 39° × 39° area of the rat retina. In the pattern bleaching experiment, the CW laser bleached an area covering 7.8° × 19.5° in dark adaptation 15 seconds before the dark-adapted OCT-scan was acquired. The OCT scans of the dark- and light-adapted states for the pattern bleach experiment covered an area of 39° × 19.5°.

### Animal procedure

We imaged albino rats (Sprague Dawley, 200–400 g, Harlan Laboratories) and wild-type pigmented rats (Long Evans, 200–400 g, Harlan Laboratories) in the study. The rats were dark-adapted for four hours before the experiment. A cocktail containing ketamine (54 mg/kg body weight) and xylazine (6 mg/kg body weight) was intraperitoneally injected for anesthesia. The rat’s pupil was dilated with 0.5% tropicamide ophthalmic solution and 0.5% proparacaine hydrochloride ophthalmic solution. After sedated, a powerless contact lens was put on the eye to prevent cornea dehydration and cataract formation^31^. To reduce movement we used a home-made animal mount to restrain the rodent and to fix the head. A bit-bar was used to fix the teeth and elastic bandage was used to fix the skull on the animal mount. All experiments were performed in compliance with the ARVO Statement for the Use of Animals in Ophthalmic and Vision Research, and approved by the Animal Care and Use Committee of Florida Internal University.

Before light adaptation all the procedures were conducted in darkroom with the help of dim red light illumination (λ > 650 nm). To avoid light exposure no computer screen was used during alignment and acquisition of the dark-adapted image. Instead, a displaying goggle similar to a virtual reality system was used for displaying all the computer operations.

## Additional Information

**How to cite this article**: Liu, T. *et al.* Depth-resolved rhodopsin molecular contrast imaging for functional assessment of photoreceptors. *Sci. Rep.* **5**, 13992; doi: 10.1038/srep13992 (2015).

## Supplementary Material

Supplementary Information

## Figures and Tables

**Table 1:** Regional pixel intensities in Fig. 4(f).

**Region of interest**	**Inner retina**		**Photoreceptor**	
	**In pre-bleach area**	**Outside pre-bleach area**	**In pre-bleach area**	**Outside pre-bleach area**
Pixel intensities (Mean ± SD)	0.0094 ± 0.0104	0.0097 ± 0.0158	0.0121 ± 0.0110	0.0885 ± 0.0575
