# Supplementary material for: Depth-resolved rhodopsin molecular contrast imaging for functional assessment of photoreceptors
Source: Sci Rep. 2015 Sep 11;5:13992. doi: 10.1038/srep13992 (PMC4566094; doi:10.1038/srep13992)
Supplement: Supplementary Information [file srep13992-s1.doc]

Depth-resolved Rhodopsin molecular contrast imaging for functional assessment of photoreceptors

Tan Liu, Rong Wen, Byron L. Lam, Carmen A. Puliafito, and Shuliang Jiao

**Supplementary Materials**

1. **Realign the A-lines in each OCT cross-sectional image as a necessary step for averaging the B-scans**


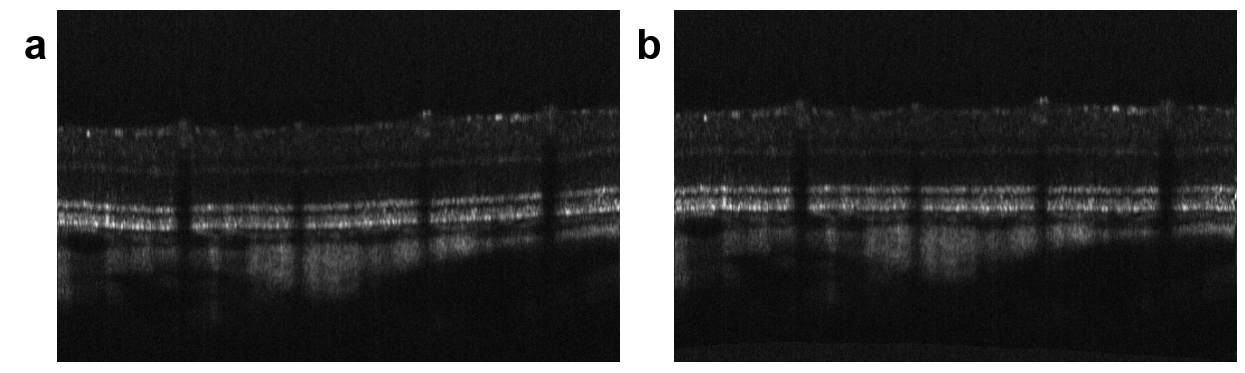


**Figure S1.** Realign the OCT cross-sectional image. (a) Original image in linear scale; (b) realigned image.

Averaging the B-scans in a 3D OCT dataset is an effective way to suppress speckle. Because of the curvature of the retina and movement of the eye during image acquisition we need to have a reference plane to align all the images before averaging is feasible. We selected the IS/OS junction as the reference plane since good alignment of the photoreceptor layer is the key for calculating the rhodopsin contrast. The two 3D OCT datasets of the dark- and light-adapted states were registered in the *x-y* plane by correlating the corresponding OCT fundus images. For each B-scan we segmented the IS/OS semi-manually and shifted each A-line in the cross sectional image in the *z* direction so that the IS/OS became a straight line, i.e. the *z* coordinate of the IS/OS in each A-line is constant in a B-scan. The aligned B-scans were then shifted in the *z* direction so that the IS/OS of the 3D dataset became a plane parallel to the *x-y* plane. Figure S1 shows a cross-sectional image before and after the alignment.

1. **Extract the local reflection information from SD-OCT cross-sectional images**

In SD-OCT, the *en face* fundus projection image can be constructed either in the time domain or the spectral domain. The projected image intensity can be expressed in the time-domain as:

$I_{t}(x,y)=F(I,R_{r},C_{fp})\sum_{\tau} \left\{2\sum_{n} \sqrt{R_{n}}\cdot\Gamma\left[\tau\pm2(\tau_{n}-\tau_{r})\right]\right\}^{2}$ B1

where *F(I, R_r_, C_fp_)* is a function of the intensity of the light (*I*), the reflectivity of the reference arm (*R_r_*), and light collecting coefficient of the sample arm (*C_fp_*), which is a constant for each OCT system and will be cancelled when the differential image is calculated using Eq. C4. *R_n_* is the normalized intensity reflection representing the contribution to the collected sample light by the *n*th scatterer. Path lengths are represented by *τ_n_* for the *n*th scatterer in the sample and *τ_r_* for the light in the reference arm. *Γ*(*τ*) is the autocorrelation function for the source light. Summation is across all the depths in the sample light beam. Therefore, the fundus image *I_t_(x, y)* can be acquired from the calculated OCT intensity signals by squaring and summing the values at all axial positions except those near *τ*=0. To extract the light intensities reflected from a certain depth range the following formula is used

$I_{t}(x,y,\tau_{1}<\tau<\tau_{2})=F(I,R_{r},C_{fp})\sum_{\tau1}^{\tau2} \left\{2\sum_{n} \sqrt{R_{n}}\cdot\Gamma\left[\tau\pm2(\tau_{n}-\tau_{r})\right]\right\}^{2}$ B2

In a spectrometer-based spectral-domain OCT system the signal or sensitivity fall-off[[1]](#endnote-2) with depth must be compensated when quantitative reflection information is extracted from the OCT signal. The fall-off characteristics was first calibrated by using two mirrors with different path-length differences. The fall-off curve was then used to compensate the pixel intensities at different depth of an OCT image.

1. **Theoretical model for the relationship of the depth resolved signals and rhodopsin concentration**

The light propagation in the retina was studied by many research groups. According to the model of fundus reflection proposed by van de Kraats *et al*,[[2]](#endnote-3) the measured light reflected from the retina is composed of reflections from three distinct layers of the eye, as summarized by Morgan *et al*.[[3]](#endnote-4) The first layer is the ocular media anterior to the rod inner segment, where wave guiding by photoreceptors begins. The second one is the photoreceptor layer, where reflection by the discs and absorption by the bleachable visual pigment occurs. The third is the post-receptor layer, including the RPE, choroid, and sclera. The intensity of the reflected light from each layer can be expressed as:

$I_{pre}=I_{0}\cdot\tau_{pre}\left(\lambda\right)^{2}\cdot\rho_{pre}\left(\lambda\right)$ C1

$I_{pr}=I_{0}\cdot\tau_{pre}\left(\lambda\right)^{2}\cdot\left[1-\rho_{pre}\left(\lambda\right)\right]^{2}\cdot COV\cdot\tau_{pr}\left(\lambda\right)^{2}\cdot\rho_{pr}\left(\lambda\right)$ C2

$\begin{matrix} I_{post}=I_{0}\cdot\tau_{pre}\left(\lambda\right)^{2}\cdot\left[1-\rho_{pre}\left(\lambda\right)\right]^{2}\cdot\{\tau_{pr}\left(\lambda\right)^{2}\cdot COV^{2}+ \\ 2\cdot\tau_{pr}\left(\lambda\right)\cdot(1-COV)+(1-COV)^{2}\}\cdot\left[1-\rho_{pr}\left(\lambda\right)\right]^{2}\cdot\tau_{post}\left(\lambda\right)^{2}\cdot\rho_{post}\left(\lambda\right) \end{matrix}$ C3

where *I_0_* is the light intensity incident into the eye, *I_pre_*, *I_pr_* and *I_post_* are the light intensities reflected from the pre-photoreceptor layer, photoreceptor layer, and post-photoreceptor layer, respectively. *τ_pre_*, *τ_pr_* and *τ_post_* are the transmittance of the three layers, respectively. *ρ_pre_*, *ρ_pr_* and *ρ_post_* are the reflectance of the three layers, respectively. *COV* is the rod photoreceptor coverage ratio on the retina.

By segmentation we can get the reflection terms from different layers. As an example, by segmenting the photoreceptor layer we can get *I_pr_*. The differential image between the light- and dark-adapted states was calculated by using:

$I(x,y)=[I_{2}(x,y)-I_{1}(x,y)]/I_{2}(x,y)$ C4

where *I*_1_ and *I*_2_ are the pixel intensity of the dark- and light-adapted images, *x* and *y* are the lateral coordinates of the images. In our model, only the *τ_pr_* is assumed to be related with the rhodopsin absorption, all the other parameters should not change before and after the bleaching. Hence, by combining the Eq. C2 and Eq. C4, we have the intensity of the differential image for the photoreceptor layer (*I_dpr_*)

$I_{dpr}(x,y)=[\left.\tau_{pr}\left(\lambda\right)^{2}\right|_{light}-\left.\tau_{pr}\left(\lambda\right)^{2}\right|_{dark}]/(\left.\tau_{pr}\left(\lambda\right)^{2}\right|_{light})$ C5

According to the Beer-Lambert law, the transmittance of the photoreceptor layer can be expressed as

$\tau_{pr}=10^{-\varepsilon\cdot c\cdot d}\cdot\tau_{non\_rho}$ C6

where *ε* is the molar extinction coefficient of rhodopsin, *c* is the rhodopsin concentration, *d* is the thickness of the outer segment, *τ_non-rho_* is the transmittance of photoreceptor layer when it’s fully bleached.

If we assume the concentration of rhodopsin under light-adapted states is zero, Eq. C5 can be expressed as

$I_{dpr}(x,y)=1-10^{-2\cdot\varepsilon\cdot c(x,y)\cdot d(x,y)}$ C7

By using Taylor Expansion, we have

$\begin{matrix} I_{dpr}(x,y)=2\cdot\ln10\cdot\varepsilon\cdot d(x,y)\cdot c(x,y)-O(c(x,y)) \\ \approx2\cdot\ln10\cdot\varepsilon\cdot d(x,y)\cdot c(x,y) \end{matrix}$ C8

where *O*(*c*) is the higher order terms which can be omitted. As a result, we can conclude that segmented differential image between the light- and dark-adapted states calculated using Eq. C4 is a representation of the distribution of rhodopsin concentration. It is obvious to see that the rhodopsin concentration is underestimated if the full depth reflection $I_{full}=I_{pre}+I_{pr}+I_{post}$is used in the calculation, the case with fundus photography or SLO based densitometry.


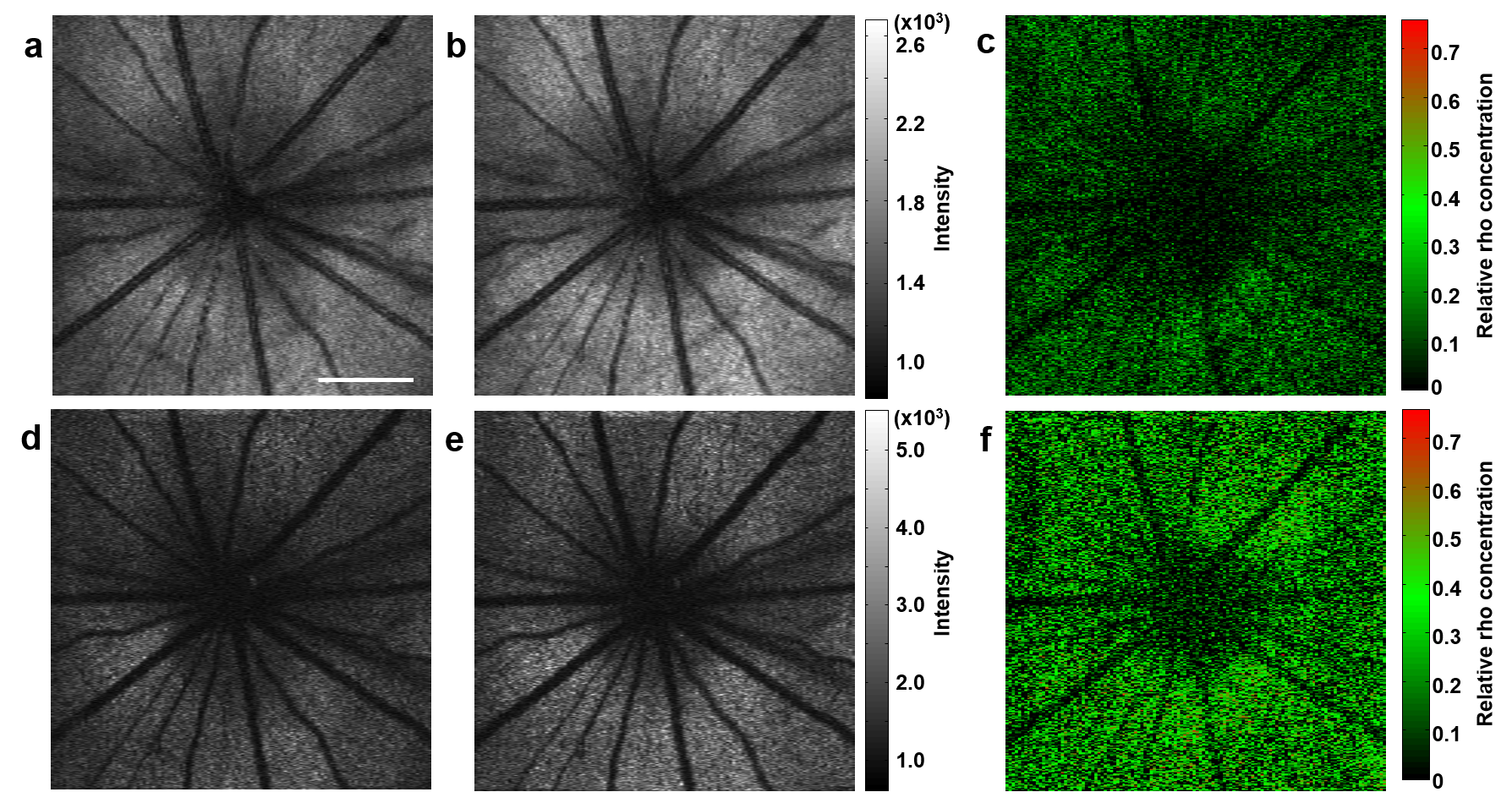


**Figure S2.** Effect of segmentation on the contrast of rhodopsin imaging. (a), (b), and (c): En face view of the dark-adapted, light-adapted, and the differential images generated with summation of the OCT signals from all the imaging depths; (d), (e), (f) En face view of the dark-adapted, light-adapted, and the differential images generated with signals from the IS/OS and the RPE only. (a) and (b), (d) and (e) share the same color map respectively. (c) and (f) share the same color map. Bar: 500 µm.

The VIS-OCT approach with depth resolution allows us to segment signals from the photoreceptor layer to construct the topographic rhodopsin distribution in the retina using the same 3D datasets of dark- and light-adapted retina in Fig. 2 in the main manuscript. Comparison between the differential images calculated from reflections of the full-depth [Fig. S2(c)] and that of only the photoreceptor and the RPE layers [Fig. S2(f)] of the retina shows that the segmented image has better imaging contrast and is a more accurate representation of rhodopsin distribution in the retina.

1. **Pattern bleaching effect**


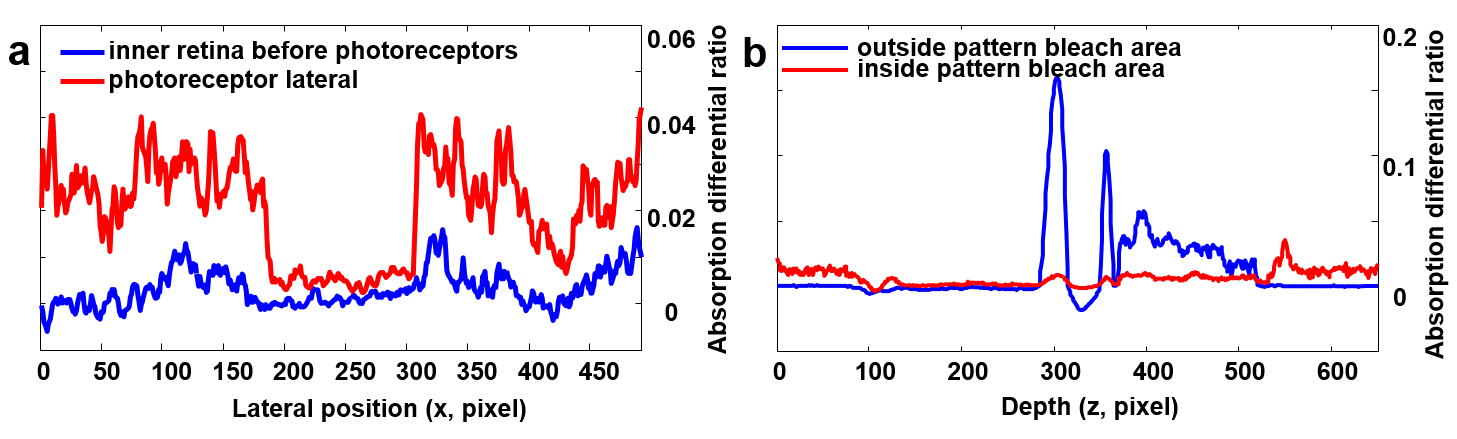


**Figure S3**. Effect of pattern bleaching. (a): Averaged pixel intensity vs lateral positions in the inner retinal area before the photoreceptor layer (blue line) and in the photoreceptor layer (red line) for the image of Fig. 4f; (h) Averaged A-lines in (red line) and outside (blue line) the pre-bleached area for the image of Fig. 4f.

1. **References**

   ?. Izatt, J. A. & Choma, M. A. Theory of optical coherence tomography. In: Drexler, W. & Fujimoto, J. editors. *Optical Coherence Tomography: Technology and Applications*, Springer New York, 47–72 (2008). [↑](#endnote-ref-2)
2. . van de Kraats, J., Berendschot T. T. & van Norren, D. The pathways of light measured in fundus reflectometry. *Vision Res*. **36**, 2229–2247 (1996). [↑](#endnote-ref-3)
3. . Morgan, J. I. & Pugh, E. N. Scanning laser ophthalmoscope measurement of local fundus reflectance and autofluorescence changes arising from rhodopsin bleaching and regeneration. *Invest Ophthalmol Vis Sci.* **54**, 2048-2059 (2013). [↑](#endnote-ref-4)
